# Right inferior frontal cortex activity correlates with tolcapone responsivity in problem and pathological gamblers

**DOI:** 10.1016/j.nicl.2016.12.022

**Published:** 2016-12-20

**Authors:** Andrew S. Kayser, Taylor Vega, Dawn Weinstein, Jan Peters, Jennifer M. Mitchell

**Affiliations:** a Department of Neurology, University of California, San Francisco, United States; b Department of Neurology, VA Northern California Health Care System, United States; c Department of Psychology, University of Cologne, Germany; d Department of Psychiatry, University of California, San Francisco, United States

**Keywords:** Gambling, Dopamine, Tolcapone, Prefrontal cortex, Ventral striatum, Frontostriatal

## Abstract

Failures of self-regulation in problem and pathological gambling (PPG) are thought to emerge from failures of top-down control, reflected neurophysiologically in a reduced capacity of prefrontal cortex to influence activity within subcortical structures. In patients with addictions, these impairments have been argued to alter evaluation of reward within dopaminergic neuromodulatory systems. Previously we demonstrated that augmenting dopamine tone in frontal cortex via use of tolcapone, an inhibitor of the dopamine-degrading enzyme catechol-O-methyltransferase (COMT), reduced delay discounting, a measure of impulsivity, in healthy subjects. To evaluate this potentially translational approach to augmenting prefrontal inhibitory control, here we hypothesized that increasing cortical dopamine tone would reduce delay discounting in PPG subjects in proportion to its ability to augment top-down control. To causally test this hypothesis, we administered the COMT inhibitor tolcapone in a randomized, double-blind, placebo-controlled, within-subject study of 17 PPG subjects who performed a delay discounting task while functional MRI images were obtained. In this subject population, we found that greater BOLD activity during the placebo condition within the right inferior frontal cortex (RIFC), a region thought to be important for inhibitory control, correlated with greater declines in impulsivity on tolcapone versus placebo. Intriguingly, connectivity between RIFC and the right striatum, and not the level of activity within RIFC itself, increased on tolcapone versus placebo. Together, these findings support the hypothesis that tolcapone-mediated increases in top-down control may reduce impulsivity in PPG subjects, a finding with potential translational relevance for gambling disorders, and for behavioral addictions in general.

## Highlights

• Nonplanning impulsivity correlates with changes in delay discounting on tolcapone.
• Inferior frontal cortical activity covaries with changes in discounting on tolcapone.
• The strength of frontostriatal connectivity correlates with tolcapone response.
• Enhancing cortical dopamine tone may be useful in treatment of addictive disorders.

## 1 Introduction

Impulsivity is a well-known correlate of addiction (Bickel et al., 2014). The tendency to choose smaller but immediate rewards over larger but delayed ones is greater in subjects with substance use disorders than in matched controls (Bickel and Marsch, 2001), and the prototypical behavioral addiction, pathological gambling, is likewise associated with steep discounting of delayed rewards (Wiehler and Peters, 2015). This increase in delay discounting has been linked to dysregulation of dopamine-based neuromodulatory systems (Volkow and Baler, 2015), which in turn have been associated with the addictive disorders themselves. For example, D2/D3 dopamine agonists are strikingly associated with the induction of problem and pathological gambling (PPG) in Parkinson's disease (Voon et al., 2009). As PET and other neuroimaging studies have begun to reveal changes both in the activation of reward circuitry (Balodis et al., 2012) and striatal dopamine measures (Joutsa et al., 2015, Linnet et al., 2011) in patients with PPG, disorders along the behavioral addiction spectrum, including PPG, are now considered to share many features with other addictions. However, because such behavioral addictions may be less confounded by use of psychoactive substances, they can potentially provide a unique opportunity to understand the role of dopamine in addictive disorders more broadly.

It has recently been suggested that the particular locus of dopamine dysregulation may be important to understanding addictive disorders (Kayser et al., 2012, Volkow and Baler, 2015), and specifically that cortical and striatal dopamine might differentially impact behaviors such as impulsivity. In part, these ideas arise from the finding that dopamine metabolism is known to be regulated differentially in the frontal cortex and striatum: while termination of dopamine's effect in the striatal synapse is primarily mediated by reuptake via the dopamine transporter, the action of synaptic dopamine in the frontal cortex is terminated primarily via degradation by the catechol-O-methyltransferase (COMT) enzyme (Chen et al., 2004, Gogos et al., 1998). We therefore reasoned that the COMT antagonist tolcapone might preferentially augment cortical dopamine tone (Tunbridge et al., 2004) and reduce impulsivity via increased activity within cognitive control regions, similar to its effects on aspects of working memory (Apud et al., 2007). In healthy controls, our previous work demonstrated that this prediction held (Kayser et al., 2012), particularly for subjects with greater baseline impulsivity as measured by the Barratt Impulsiveness Scale (BIS). Similarly, an open-label study of tolcapone without a placebo control in patients with gambling disorders suggested that changes in frontoparietal brain activity during performance of a Tower of London task (a task to assess planning) on tolcapone correlated with changes in patients' scores on the Yale Brown Obsessive Compulsive Scale Modified for Pathological Gambling (PG-YBOCS) across time (Grant et al., 2013). Conversely, Pine and colleagues demonstrated that healthy subjects given the dopamine precursor L-dopa, which should act throughout the brain, showed consistent increases in delay discounting (Pine et al., 2010). This distinction between frontal and striatal dopamine, possibly due to their time courses (tonic versus phasic, respectively) or their competing influences on frontostriatal “top-down” circuitry, has been suggested to define a potential mechanism for biasing decisions toward later versus sooner choices (Volkow and Baler, 2015).

Complicating the above is the importance of individual differences, and the related knowledge that PPG and other addictive disorders are very likely syndromic – i.e. that diverse etiologies may give rise to a common phenotype that is unlikely to respond in the same manner to a given intervention. Efforts to define a vulnerability phenotype may therefore help to predict treatment response, in keeping with increasing clinical interest in “precision” (or personalized) medicine (Jameson and Longo, 2015). Previous work has argued for the importance of neural phenotypes in particular, with candidate regions derived from cognitive neuroscience research (Ekhtiari et al., 2016). For PPG, putative neural signatures have been identified in reward-related structures including the nucleus accumbens and striatum, as well as in frontal regions thought to be important for valuation (e.g. ventromedial prefrontal cortex) and cognitive control (lateral prefrontal cortex) (Potenza, 2014).

Here we sought to evaluate individual differences in, and potential neural correlates for, the response of PPG subjects to tolcapone. Using reductions in impulsive choice on a delay discounting task as a behavioral assay, we reasoned that specific subjects who demonstrated such reductions would be sensitive to medication-induced increases in cortical dopamine tone. Such sensitivity would be accompanied by changes in the function of prefrontal cognitive control regions, which should consequently exert greater influence over subcortical structures. We thus hypothesized that tolcapone response should correlate with activity within cognitive control regions of the lateral frontal cortex, and that the connectivity of these lateral frontal areas with subcortical structures should increase in proportion to the reduction in delay discounting.

## 2 Materials and methods

### 2.1 Subject population

**Fig. 1:**
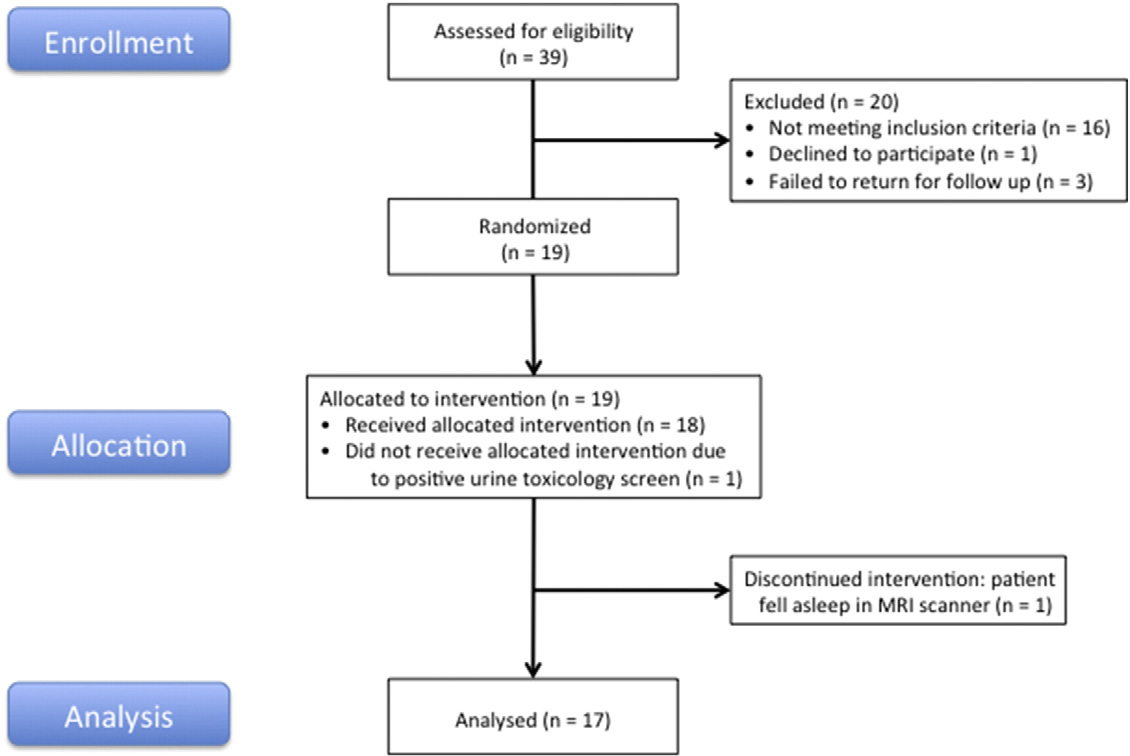
Study Flow Diagram. As documented in Materials and methods, 39 subjects were screened, of whom 19 met criteria for study participation and were allocated to the intervention. During participation in the randomized, double-blind, placebo-controlled, within-subject portion of the study, two additional subjects were excluded: one after he failed a urine toxicology screen at his first MRI visit, and another after she fell asleep during her second fMRI session.

Using advertisements placed via a community-based recruitment tool (Craigslist), we screened 39 subjects, 19 of whom were found to have South Oaks Gambling Scale (SOGS) scores ≥ 5 (mean 10.5 ± 3.4 (sd), range 6–18) as well as no history of medical, psychiatric, or neurological contraindications, and were therefore eligible to participate in the study (Fig. 1). Two subjects were subsequently excluded: one after he failed a urine toxicology screen at his first MRI visit, and another after she fell asleep during her second fMRI session. All subjects gave written informed consent in accordance with the Declaration of Helsinki and the Committee for the Protection of Human Subjects at the University of California, San Francisco and University of California, Berkeley; they were compensated for their participation. Ages ranged from 20 to 47 years old (31.5 ± 8.9 (sd)); 6 of 17 were female (Table 1). Subjects first underwent a history and physical exam, as well as blood testing for liver function and urine screening for drugs of abuse (see below), to ensure that there were no medical contraindications to tolcapone use or MRI scanning. All subjects had normal neuroanatomy as reviewed by a neurologist (A.S.K.), were right-handed, and had normal or corrected-to-normal vision. Before scan sessions, subjects were briefly trained on the delay discounting task in order to familiarize them with task procedures. Subjects then underwent two separate 1.5-h fMRI sessions, each consisting of 6 task runs of 33 trials each for a total of 198 trials, along with one resting state run (which was not further evaluated in this study). Each of the 6 task runs lasted approximately 9 min, with breaks in between to reduce fatigue.

In addition to the requirements for gambling behavior as assessed by the SOGS, inclusion criteria required that subjects be between 18 and 50 years old, right-handed, in generally good health, able to read and speak English, and able to provide informed consent. Women of reproductive age were required to be using an effective form of contraception, and to be neither pregnant nor lactating during study participation. Subjects were excluded if they demonstrated a positive urine drug toxicology screen before any visit, showed an alcohol level greater than zero as measured by breathalyzer before any visit, or reported using psychoactive substances (including both prescription medications and drugs of abuse) within the prior two weeks, or drugs of abuse more than ten times in the previous year. In addition, subjects with a current dependence on marijuana, or who had experienced any previous medical complications of marijuana use, were not eligible; otherwise, subjects could use marijuana no more than three times per week and were required to refrain from marijuana use for at least 48 h prior to testing sessions. These criteria did not apply to nicotine; the two subjects who were regular smokers were both easily able to refrain for the duration of MRI scanning and otherwise continued their regular use. Subjects who were taking medications with dopaminergic, serotonergic, or noradrenergic actions (although animal work suggests that tolcapone induces increases in dopaminergic but not noradrenergic concentrations (Tunbridge et al., 2004)), or who had a known allergy to either tolcapone or the inert constituents in tolcapone capsules, were also excluded. Similarly, after completion of the Mini International Neuropsychiatric Interview (Sheehan et al., 1998), subjects who met screening criteria for an Axis I psychiatric disorder other than gambling disorder, such as major depression, or who had a significant medical or psychiatric illness requiring treatment, were excluded from participating. Because tolcapone carries the potential for hepatotoxicity, liver function tests were required to be no more than three times the upper limit of normal. Finally, subjects were required to be free of MRI contraindications.

Using a random number generator, one of the authors (J.M.M.) randomized consecutive subjects to receive either placebo or tolcapone on their first session, and the other treatment on their second session. Blinded drug assignments were listed as either “A” or “B”. Beyond the planning of the study, J.M.M. did not otherwise participate until she contributed to writing the manuscript once the blind had been broken at study completion. All other authors of the paper, as well as the subjects, were blinded to study drug assignments throughout. Because tolcapone might discolor the urine (and therefore might inadvertently unmask drug assignments), the B-vitamin riboflavin was added to both tolcapone and placebo capsules in order to conceal this effect.

### 2.2 Sample size and randomization

Power analyses for fMRI studies rely upon assumptions about BOLD signal amplitude, smoothness, brain location, and other factors that render principled a priori designations difficult. Based upon empirical, systematic MRI analyses indicating that fMRI studies generally reach good replication at approximately 20 subjects (Desmond and Glover, 2002, Thirion et al., 2007), we targeted this number of participants. Given the challenges inherent in studying this patient population, as well as the financial and temporal constraints of pharmacological fMRI studies, our recruitment was terminated after 19 subjects had been enrolled. Subjects were recruited between August 2014 and October 2015.

### 2.3 Experimental paradigm

**Fig. 2:**
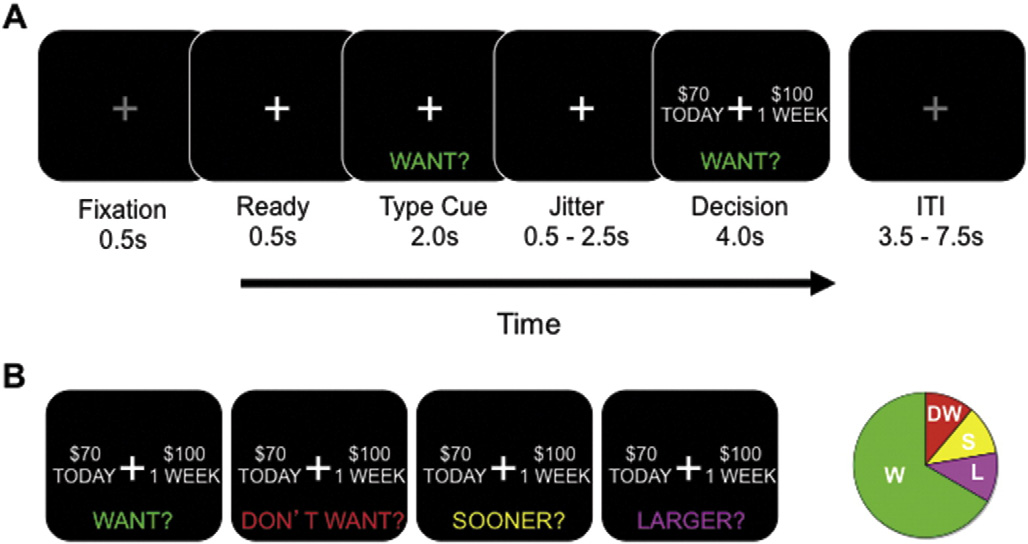
Task Design. A. Each trial of the delay discounting task began with fixation, followed by a cue to the trial type. After a brief jittered delay, subjects were prompted to make a decision (in this case, a “Want” decision). B. Illustrated are the four trial types: Want, Don't Want, Sooner, and Larger (see Materials and methods). The pie chart at right illustrates the relative proportions of each of the trial types.

Subjects were randomized in double-blind, counterbalanced, placebo-controlled fashion to either placebo or a single 200 mg dose of tolcapone on their first visit and the alternative treatment on their second visit. This dose was based upon our previously published findings that a single 200 mg dose has measurable behavioral effects (Kayser et al., 2012, Kayser et al., 2015, Saez et al., 2015). After receiving task instructions and undergoing a brief practice session of 10–20 trials, subjects performed a delay discounting task (Fig. 2) within the MRI scanner while blood-oxygen level dependent (BOLD) images were obtained. This task was chosen because, although subjects engaged in a variety of gambling-related activities (Table 1), delay discounting is thought to reflect a vulnerability to addiction that crosses multiple addiction subtypes (Bickel et al., 2014). Subjects made a button press to select one of the two presented options in the delay discounting task, randomly assigned to the left and right sides of the screen. The “Later” option consisted of five amounts ($5, $10, $20, $50, or $100) at one of five future delays (1 week, 2 weeks, 1 month, 3 months, or 6 months). The percentage difference between the Now and Later options was selected from one of four different values (50%, 30%, 15%, and 5%). Subjects entered the MRI scanner 90 min after drug ingestion to ensure that the delay discounting task was performed while drug levels were presumably at peak (approximately 120 min, per tolcapone package insert (Valeant Pharmaceuticals) and pharmacokinetic studies (Whelan et al., 2012)). The 198 task trials for each session were presented in pseudorandom order. No other tasks were administered in the MRI scanner.

At the start of each trial, subjects were cued to one of four trial types: “Want”, “Don't Want”, “Sooner”, and “Larger” (Fig. 2). For each of these trial types, subjects were then presented with two hypothetical alternatives: a smaller amount of money available today (“Now”) and a larger amount available at a future point in time (“Later”). We have previously shown that this paradigm with hypothetical rewards effectively engages subjects (Kayser et al., 2012), consistent with reports that hypothetical rewards activate common brain regions involved in value computations (Bickel et al., 2009, Kang et al., 2011). Each of the four trial types defined in the task allowed us to investigate different functions. In the Want condition, the primary analytic focus of this study, subjects chose the option they preferred. In the Don't Want condition, subjects also chose the option they preferred, but then made a button-press to select the opposite choice. This condition permitted us to evaluate motor impulsivity (Mitchell et al., 2005). In the Sooner and Larger conditions, which we combined to form a control condition, subjects simply selected the sooner or larger options, respectively. These trial types allowed us to ensure that subjects were appropriately following instructions, and to introduce a condition in which the decision about monetary options was not a motivated choice. The Want condition comprised 67% of all trials; the control conditions comprised 22%; and the Don't Want condition comprised the remaining 11%. As expected, subjects performed very well in the control condition (accuracy = 0.95 ± 0.02 (sem)) and therefore we do not further report results of the control condition in this paper.

The primary behavioral outcome was the impulsive choice ratio (ICR), which represents the ratio of the number of sooner choices to the number of total choices in the Want condition. ICR values underwent an arcsine-square root transform – i.e. were variance-stabilized – to permit the application of parametric statistical tests. Additionally, we calculated a number of related measures of impulsive choice, including a measure of the hyperbolic discounting rate (*k*). Specifically, we calculated *k* for each delay D using the cumulative dollar ratio (CDR: i.e. the ratio of all dollar amounts chosen to the cumulative maximum dollar amount available for that delay (Mazur, 1987)) and averaged across all values, as in our previous work (Mitchell et al., 2005) - i.e. CDR = 1/(1 + *k*D). This formula permitted us to define *k* even though our choice of monetary amounts, delays, and monetary differences was not necessarily optimized to define an indifference point. Using this approximation, we found that the hyperbolic discounting rate was highly correlated with ICR (e.g. r (ICR, *k*) = 0.83, p = 0.00004), as it was for changes on tolcapone versus placebo (r(ΔICR, Δ*k)* = 0.63, p = 0.0064). Thus, we elected to study ICR, given its simple and intuitive qualities (Mitchell et al., 2005). However, because the hyperbolic measure *k* can serve to link findings across different discounting values, paradigms, and studies, we made use of it when exploring cross-study comparisons, as in the Discussion.

### 2.4 Experimental paradigm: ancillary testing

At the screening visit, subjects also completed a questionnaire to assess impulsivity, the Barratt Impulsiveness Scale (BIS) (Patton et al., 1995). In addition to providing a validated impulsivity measure that was independent of the delay discounting measure, its subdivision into three primary factors – motor, attentional, and non-planning – permitted us to more specifically investigate (in this case) non-planning impulsivity. Given that this factorization of the BIS has been replicated by some (Spinella, 2007) but not by all studies (Freemantle et al., 2007, Morean et al., 2014, Reise et al., 2013), we also evaluated the two factor division of the BIS into a cognitive and a behavioral factor as defined by Reise and colleagues (Reise et al., 2013).

Additionally, both before drug administration and after the scanner run, subjects completed a speeded responding task to assess potential changes in motor function on and off tolcapone. Subjects were required to make a button press response as soon as possible after the presentation of either a brief visual or auditory stimulus; reaction times were compared both within each session and across the tolcapone and placebo conditions. In keeping with the use of this potentially vasoactive medication, subjects' blood pressures were recorded and compared both before and approximately 2.5 h after tolcapone and placebo ingestion. No subjects reported potential side effects under either the placebo or tolcapone conditions.

### 2.5 MRI image acquisition & preprocessing

MRI scanning was conducted on a Siemens MAGNETOM Trio 3 T MR Scanner at the Henry H. Wheeler, Jr. Brain Imaging Center at the University of California, Berkeley. Anatomical images consisted of 160 slices acquired using a T1-weighted MP-RAGE protocol (TR = 2300 ms, TE = 2.98 ms, FOV = 256 mm, matrix size = 256 × 256, voxel size = 1 mm^3^). Functional images consisted of 24 slices acquired with a gradient echoplanar imaging protocol (TR = 1370 ms, TE = 27 ms, FOV = 225 mm, matrix size = 96 × 96, voxel size = 2.3 × 2.3 × 3.5 mm). A projector (Avotec SV-6011, http://www.avotecinc.com, Stuart, Florida) was used to display the image on a translucent screen placed within the scanner bore behind the head coil. A mirror was used to allow the subject to see the display. Subjects made their responses via an MRI-safe fiber optic response pad (Inline Model HH-1 × 4-L, http://www.crsltd.com, Rochester, Kent, UK).

### 2.6 fMRI preprocessing and data quality assurances

fMRI preprocessing was performed using both the AFNI (http://afni.nimh.nih.gov) and FSL (http://www.fmrib.ox.ac.uk/fsl) software packages. Functional images were converted to 4D NIfTI format and corrected for slice-timing offsets. Motion correction was carried out using the AFNI program *3dvolreg*, with the reference volume set to the mean image of the first run in the series. Images were then smoothed with a 5 mm FWHM Gaussian kernel. Co-registration was performed with the AFNI program *3dAllineate* using the local Pearson correlation cost function optimized for fMRI-to-MRI structural alignment. The subsequent inverse transformation was used to warp the anatomical image to the functional image space. Anatomical and functional images were then normalized to a standard volume (MNI_N27: 3 mm × 3 mm × 3 mm voxels) using the FSL program *fnirt* available from the Montreal Neurological Institute (MNI; http://www.bic.mni.mcgill.ca) prior to application of univariate and other tests. Measures related to movement and image quality were inspected for every run, and any runs that were contaminated by movement > 2.5 mm or an excessive number of outlier voxels as defined by the AfNI function *3dToutcount* were removed. Our imaging protocol did not include a dedicated scan to assess magnetic field homogeneity.

### 2.7 Univariate analysis

To address a series of hypotheses, we carried out a number of voxel-wise fMRI statistical analyses for each subject using the general linear model (GLM) framework implemented in the AFNI program 3*dDeconvolve*. The BOLD correlates of different decisions were assessed by modeling each of the cue and decision phases of the task for the four different task conditions (Want, Don't Want, Sooner, Larger) with separate regressors, each of which was derived by convolving a gamma probability density function (peaking at 6 s) with a vector of stimulus onsets for each condition. Subsequent univariate analyses evaluated individual conditions (e.g. Want, Don't Want) during the decision phase (Fig. 2). In addition, every GLM analysis reported in the manuscript included regressors of no interest: specifically, the 6 motion regressors, and terms for zero through fourth order signal drift. Map-wise significance (p < 0.05, corrected for multiple comparisons) was determined by applying a cluster-size correction derived from the AFNI programs *3dFWHMx* and *3dClustSim* on data initially thresholded at a value of p < 0.005 (uncorrected). Because cortical dopamine projections are predominantly frontal (Cools, 2008), univariate analyses addressed more specific task-related hypotheses about changes in frontostriatal regions by using the AAL template brain (Tzourio-Mazoyer et al., 2002) to generate a frontostriatal mask (AAL areas 3–32 and 71–76). Given the above constraints, the appropriate cluster size correction was determined to be 29 voxels for these analyses. For the analysis of the main effect of task (Supplementary Information), we evaluated the whole brain. (This main effect was defined as activity during all phases and trial types of the task for the tolcapone session compared to the tolcapone-specific baseline, minus the corresponding comparison for the placebo session.) To achieve a corrected map-wise significance of p < 0.05 for this contrast, the appropriate cluster size correction was determined to be 44 voxels for data initially thresholded at p < 0.005, uncorrected.

### 2.8 Connectivity analysis

In order to evaluate connectivity between seed regions and other brain areas, we performed a generalized psychophysiological interaction (PPI) analysis (McLaren et al., 2012). We first extracted the time series from the region of the right inferior frontal cortex defined by the univariate analysis. After deconvolution, interaction regressors were defined independently for the Want condition in the placebo and tolcapone runs, which were then contrasted to determine changes in connectivity between the two conditions. To address our hypothesis about tolcapone-induced changes in frontostriatal connectivity, we used the AFNI programs *3dFWHMx* and *3dClustSim* and the AAL-defined regions for the right striatum (72 and 74) to define the appropriate small volume cluster size correction (10 voxels) for an uncorrected p-value of 0.005, resulting in a significance level of p < 0.05 (corrected). Only the right striatum was selected because of emerging, albeit limited, evidence that the right inferior frontal cortex is more strongly connected to the right basal ganglia (specifically, to the right subthalamic nucleus) compared to the left (Forstmann et al., 2010), and that stimulation of the right inferior frontal cortex changes activity within the right but not the left striatum (Zandbelt et al., 2013).

### 2.9 Statistical analysis

For analysis of behavioral data, *t*-tests and Pearson's correlation coefficients were used to calculate statistical significance. Qualitative estimates of effect size were applied based on the work of Cohen (Cohen, 1988). For univariate and connectivity analyses of BOLD data, significance was calculated using statistical techniques and corrections implemented in the AFNI software package, including the functions *3dDeconvolve*, *3dFWHMx*, *3dClustSim*, and *3dttest ++.*

## 3 Results

**Fig. 3:**
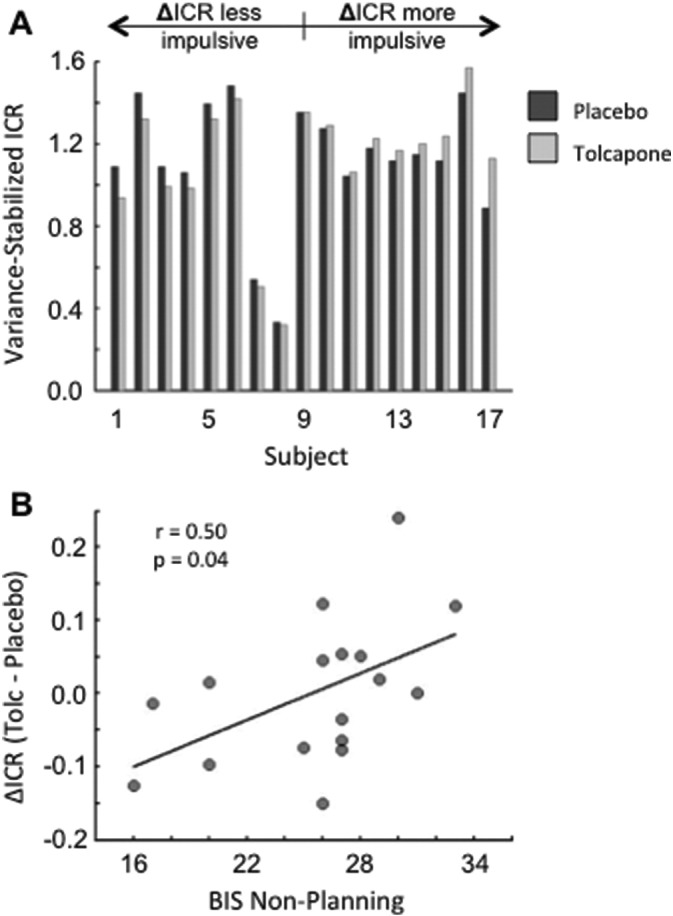
Behavioral Results. A. Shown are the variance-stabilized impulsive choice ratios for each subject in the placebo (dark gray) and tolcapone (light gray) conditions, ordered by magnitude of the difference within the 17 subjects. Subjects whose ICR values decreased on tolcapone are to the left. B. The change in ICR on tolcapone versus placebo was significantly correlated with subjects' scores on the non-planning subscale of the Barratt Impulsiveness Scale (BIS).

Consistent with our hypotheses, subjects exhibited impulsive choice ratios (ICRs) in which they chose significantly more sooner than later options (T(16) = 4.49, p = 0.00037; Fig. 3A). Across all subjects, there was no significant difference in ICR on tolcapone versus placebo (t(16) = 0.075, p = 0.94 (ns)). However, the change in ICR for individual subjects was significantly correlated with the non-planning subscale of the Barratt Impulsiveness Scale (BIS: r = 0.50 (r^2^ = 0.25), p = 0.04; medium to large effect size (Cohen, 1988); Fig. 3B), though not with the motor or attention subscales (p > 0.38 (ns)). Given that this factorization of the BIS has been replicated by some (Spinella, 2007) but not by all studies (Freemantle et al., 2007, Morean et al., 2014, Reise et al., 2013), we repeated these correlations using the two factor approach to the BIS of Reise and colleagues (Reise et al., 2013). Consistent with the above results, a trend-level correlation could be seen with the unweighted cognitive impulsivity factor (r = 0.44, p = 0.075) but no relationship was seen with a behavioral impulsivity factor (r = 0.0, p = 0.99 (ns)). In a separate analysis, no relationship was seen between ΔICR and the South Oaks Gambling Scale (SOGS) (r = − 0.1, p = 0.7 (ns)). Additionally, when *Z*-scored and averaged totals for the PPG-related SOGS, GRCS, and GSAS (placebo) questionnaires (Table 1) were summed and correlated with ΔICR, no relationship was seen (r = − 0.25, p = 0.34 (ns)).

Importantly, the changes in ICR across subjects were not due to nonspecific dopaminergic effects on motor responding. Preferences for sooner versus later choices in the Don't Want condition, a test for motor impulsivity, correlated strongly with those in the Want condition (r = 0.85 (r^2^ = 0.72), p ≪ 10^− 5^; large effect size) across both drug conditions. Moreover, in a simple speeded response task, no difference was seen in reaction time for either visual or auditory responding on tolcapone compared to placebo (all T(14) ≤ 1.1, p ≥ 0.3), and there was no correlation across individuals between changes in motor responding and changes in ICR on tolcapone (all | r | values ≤ 0.11, p ≥ 0.7). Finally, no differential changes in blood pressure were seen on tolcapone versus placebo (T(15) = 1.31, p = 0.21), and subjects themselves were unable to distinguish between tolcapone and placebo administration based on confidence ratings (T(16) = 0.98, p = 0.34).

**Fig. 4:**
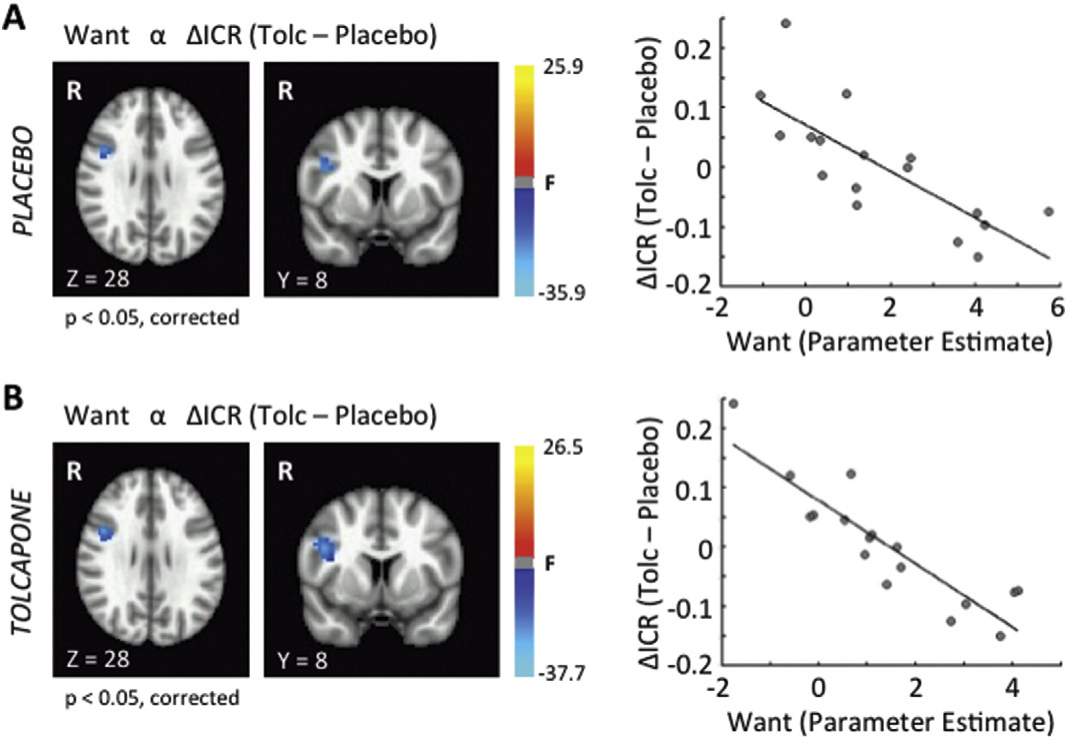
Brain-Behavior Correlation. A. Two brain regions demonstrated significant negative correlations between ΔICR and BOLD activity during the Want condition on placebo at a significance level of p < 0.05, corrected: a region within the right inferior frontal cortex (shown in axial and coronal slices) and a more dorsal, posterior region in the right premotor cortex (Table 2). Greater BOLD signal in these regions covaried with greater declines in ICR on tolcapone versus placebo. Shown in the right panel are the parameter estimates across subjects for the region within the right inferior frontal cortex, demonstrating that these effects were not driven by outlier values (for reference, the equivalent Pearson's r = − 0.77 (r^2^ = 0.59)). A similar result was seen for the right premotor cortex (data not shown). B. This finding was replicated in the tolcapone condition. The same two brain regions demonstrated significant negative correlations between ΔICR and BOLD activity during Want trials at a significance level of p < 0.05, corrected (left panel); and greater BOLD signal in these regions again covaried with greater declines in ICR on tolcapone versus placebo (right panel, shown for the RIFC region; for reference, the equivalent Pearson's r = − 0.89 (r^2^ = 0.79)).

We next searched for neural correlates of the tolcapone response. Consistent with the absence of a group-level behavioral effect of tolcapone, minimal changes were noted in group-level prefrontal cortical activity within a contrast of the main effect of task for tolcapone versus placebo (see Supplementary Information). Notably, however, individual differences in behaviorally relevant prefrontal activity were seen. To identify correlates of the tolcapone response, we first correlated the change in ICR on tolcapone versus placebo with BOLD activity during Want trials in the placebo condition. This analysis identified two regions in the right prefrontal cortex (p < 0.05, corrected) whose activity correlated inversely with the change in ICR on tolcapone: a right premotor region (Table 2), and an area in the right inferior frontal cortex (RIFC: Fig. 4A and Table 2) that has previously been associated with inhibitory control (Aron et al., 2014, Hampshire and Sharp, 2015) and the use of illicit substances (Whelan et al., 2012). To ensure that this relationship in RIFC was not artifactual, we first determined that it was not driven by outlier subjects (Fig. 4A, right panel). In addition, when we correlated the change in ICR on tolcapone versus placebo with BOLD activity during Want trials in the tolcapone, rather than the placebo, condition, we were able to replicate our result from the placebo condition (Fig. 4B); and when we used this area for a region of interest analysis for Don't Want trials, we also identified a significant negative correlation between the change in ICR and activity within the RIFC across subjects (r = − 0.64 (r^2^ = 0.41), p = 0.006 (placebo); r = − 0.69 (r^2^ = 0.48), p = 0.002 (tolcapone)) that approximated the large effect sizes seen in Fig. 4. Importantly, our results were also independent of the specific delay discounting measure. Although the study was designed to assess ICR, *k* values obtained from a hyperbolic discounting function determined from each subject's data also correlated strongly with RIFC activity. Specifically, Δ*k* and BOLD activity within the RIFC region of interest were strongly and inversely correlated in both the placebo (r = − 0.73 (r^2^ = 0.53), p = 0.0009) and tolcapone (r = − 0.68 (r^2^ = 0.46), p = 0.003) conditions, consistent with the high percentage of shared variance in the ΔICR and Δ*k* values (see Materials & Methods). Taken together, these findings are reassuring, in that the potential importance of the RIFC is not isolated to a single drug condition, a single decision type (Want or Don't Want), or a single delay discounting measure (ICR or *k*). In particular, the fact that we can replicate this finding ameliorates recent concerns about the appropriateness of cluster size corrections in multiple neuroimaging packages (Eklund et al., 2016). However, these results also indicate that activity in this region does not itself change with tolcapone, raising a question about how RIFC influences decision making in response to tolcapone administration.

**Fig. 5:**
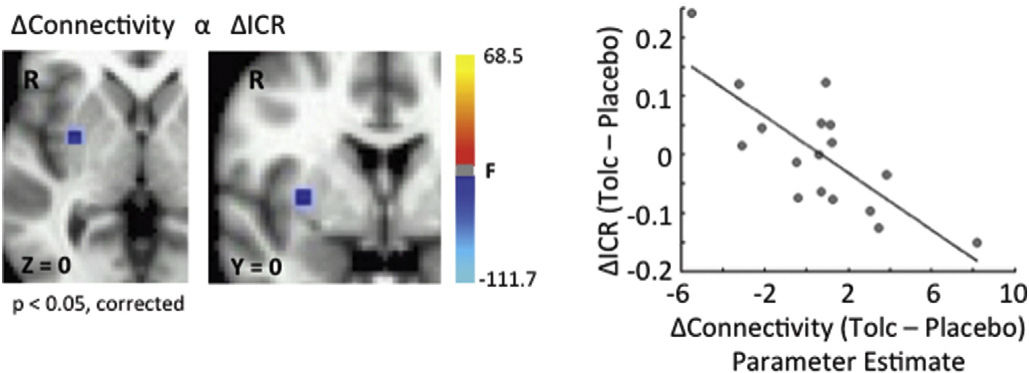
Connectivity-Behavior Correlation. A psychophysiological interaction (PPI) analysis was performed in which the right inferior frontal cortex (RIFC) served as the seed region and the right striatum as the search volume of interest. Shown is the correlation between connectivity with the RIFC during the Want condition (tolcapone versus placebo) and the change in ICR (tolcapone versus placebo), thresholded at a significance level of p < 0.05, corrected (right panel; for reference, the equivalent Pearson's r = − 0.76 (r^2^ = 0.58)). Specifically, greater increases in RIFC ↔ right putamen connection strength on tolcapone versus placebo correlated with greater declines in ICR on tolcapone versus placebo (left panel). When the search was expanded to the rest of the brain, no other regions of this cluster size or greater were found (data not shown).

We reasoned that while activity within the RIFC may not change, its ability to influence other regions – i.e. its connectivity – might differ across drug conditions. To evaluate this possibility, we conducted a psychophysiological interaction (PPI) analysis to search for differences in corticostriatal connectivity. We used the RIFC as a seed (MNI coordinates 43, − 8, 28; see Fig. 4 and Table 2), and directly compared its connectivity with the right striatum during Want decisions on tolcapone versus placebo. This analysis identified a single 10-voxel cluster in the right putamen (p < 0.05, corrected; Fig. 5) whose connectivity with the RIFC increased to a greater extent on tolcapone in those subjects whose ICR declined more strongly on drug. In other words, greater increases in RIFC-right putamen connectivity on tolcapone correlated with greater declines in ICR on tolcapone. Importantly, when we expanded our search space to the whole brain, no clusters of this size or greater were identified elsewhere, suggesting that this change in connectivity with ΔICR may be specific to this corticostriatal connection – i.e. similar changes could not be found with regions of interest of this size or greater.

## 4 Discussion

Here we demonstrate that subjects with PPG responded differentially to a medication, tolcapone, that augments frontal dopamine tone. More intriguingly, the change in ICR on tolcapone correlated with subjects' scores on the non-planning subscale of the BIS, and greater reductions in ICR covaried with greater activity within the right inferior frontal cortex (RIFC). However, the level of activity within the RIFC did not itself differentiate the placebo from the tolcapone conditions; rather, as compared to placebo, tolcapone increased functional connectivity of the RIFC with the right striatum. Together these results suggest that tolcapone may work most effectively in those subjects with PPG who show greater RIFC activity at baseline, and that future studies might determine whether activity in these regions has the potential to serve as a useful biomarker for the therapeutic efficacy of tolcapone.

Of interest, these findings in subjects with PPG differ from our previous findings using this same task and medication in healthy control subjects (Kayser et al., 2012), where reductions in impulsivity on tolcapone correlated with greater, rather than lesser, baseline scores on the BIS scale or its non-planning subscale. One possible explanation relates to the fact that the PPG population here was significantly more impulsive than those healthy subjects in our past study (Kayser et al., 2012) in both the placebo (Z = 2.8, p = 0.0044) and the tolcapone (Z = 3.4, p = 0.000076) conditions by median k values (Placebo: 0.0017 versus 0.0051; tolcapone: 0.000094 versus 0.0074). The impact of baseline dopamine tone on dopamine response – i.e. the well-known inverted U-shaped dopamine response (Cools and D'Esposito, 2011) – would suggest that the response to a dopaminergic agent should depend nonlinearly on dopamine-sensitive functions such as baseline impulsivity (Cools et al., 2007). This hypothesis would be supported by at least one previous result: Clark and colleagues demonstrated that negative urgency - i.e. impulsivity related to negative mood states - varied in U-shaped fashion with the binding potential of the D2-receptor antagonist raclopride in the limbic striatum for a study population consisting of both controls and pathological gamblers (Clark et al., 2012). When we combined subjects from the current study with our previous study of control subjects to test the idea that a U-shaped relationship might exist between tolcapone response and non-planning impulsivity, a second-order polynomial did indeed provide a better fit to the data than either first or higher-order polynomials that modeled Δ*k* versus non-planning impulsivity; but the second-order fit did not reach significance (F(2.37) = 2.04, p = 0.14 (ns)). Future work to increase subject numbers may be worthwhile to evaluate whether this more general finding holds.

In another difference from our previous study, here we identified a neural correlate of ΔICR in a presumptive inhibitory control region, the RIFC, where previously we had found ΔICR-related activity in the left insula and left putamen in control subjects. In both cases, the relationship between ΔICR and the imaging data was confirmed by consistent data in other task conditions, arguing against artifact. One possible explanation is that activity in the RIFC is involved in the development or maintenance of addictive behaviors. The RIFC has elsewhere been shown to play a pivotal role in inhibitory control; specifically, it is commonly activated when subjects perform tasks requiring intermittent unexpected inhibition of planned actions, as in the stop signal paradigm (reviewed in (Aron et al., 2014)). While controversy continues to exist about the cognitive process instantiated by this region – i.e. whether it implements an inhibitory “stop signal” itself or whether it is part of a larger network that provides a more general monitoring function (Hampshire and Sharp, 2015) – its activity has nonetheless previously been linked to addictive disorders. Loss of gray matter in closely adjacent regions has been found in PPG patients relative to control subjects (Mohammadi et al., 2015), and patients with a history of methamphetamine addiction similarly show selective atrophy within this brain area (Tabibnia et al., 2011). Moreover, in a large study of adolescents, activity within the RIFC during a stop signal task strongly differentiated those subjects who had used alcohol, nicotine, and at least one illicit substance from those who had not (Whelan et al., 2012). Because RIFC activity in this study was elevated only for the subjects with the highest substance use burden, the authors suggested that this increased signal represented compensation – i.e. that subjects at higher risk of substance use disorders required greater activity in this region to implement the same inhibitory control as subjects without a substance use history. That interpretation would be consistent with our current findings, in that greater baseline RIFC activity was correlated with greater behavioral response to tolcapone. More generally, these data potentially argue that one cannot easily extrapolate from a control to a patient population – i.e. that these populations are qualitatively different, in that the neural underpinnings of delay discounting vary depending upon the presence of inhibitory control deficits. Nonetheless, further work will be necessary to confirm or refute such activity differences between PPG subjects and controls.

Likewise, the above results are broadly consistent with the idea that remediating impulsive behavior in patients with PPG may depend on treatments that normalize function within dopamine-sensitive brain systems (Zack and Poulos, 2009). Although ongoing work to identify potential genetic contributions to delay discounting in PPG has failed to replicate loci linked to delay discounting, including the functional COMT rs4680 (Val158Met) polymorphism, a combination of multiple dopamine genes may account for up to 17% of discounting variance in subjects with PPG (Gray and MacKillop, 2014). A PET study has further demonstrated that greater temporal discounting covaries with both decreased ventral striatal binding potential and reduced dopamine release to large rewards (Joutsa et al., 2015). Perhaps most relevant to the current work, one previous study has investigated a potential role for tolcapone in treatment of PPG (Grant et al., 2013). In this open-label study, Grant and colleagues evaluated the effects of tolcapone on performance of a Tower of London task (a task to assess planning) both before and after tolcapone treatment while obtaining functional MRI data. Using a combined frontal and parietal region of interest obtained from healthy subjects, they showed that activation increased from pre- to post-study in patients with a history of pathological gambling and correlated with changes in scores on the Yale Brown Obsessive Compulsive Scale Modified for Pathological Gambling (PG-YBOCS). Although this study did not have a corresponding placebo control, it nevertheless demonstrated the safety of tolcapone and, consistent with the results reported here, documented potential effects of the medication on activity within frontoparietal brain regions in patients with pathological gambling.

Our findings are also consistent with the idea that failures of prefrontally mediated top-down inhibitory control predispose to addiction phenotypes (Everitt and Robbins, 2016). In keeping with earlier hypotheses, we found that subjects who responded to tolcapone with decreases in impulsive choice did not show differences in RIFC activity on tolcapone versus placebo, but instead experienced corresponding changes in frontostriatal function - specifically, increases in the connectivity of the RIFC with the R putamen. Such changes in cognitive control and connectivity, in the absence of localized activity increases, could be implemented by increases in the synchrony between remote brain areas (Voytek et al., 2015). More generally, the idea that increases in such frontostriatal connectivity need not be dependent upon changes in localized RIFC activity to improve impulsivity (at least, as measured by delay discounting behavior) is consistent with the general idea that self-regulation is implemented via top-down, frontostriatal mechanisms (Heatherton and Wagner, 2011). In PPG in particular, previous work has demonstrated that such subjects may have impairments in frontostriatal activity: for example, during a paradigm that involved wins, losses, and near misses, van Holst and colleagues showed that ventral striatal connectivity with the insula in patients with pathological gambling was stronger for near misses than for full misses (van Holst et al., 2014). In contrast, Balodis and colleagues did find declines in univariate activity within the ventromedial PFC and ventral striatum, relative to controls, when subjects performed the Monetary Incentive Delay (MID) task (Balodis et al., 2012), though functional connectivity metrics were not evaluated. Both of these reports are potentially consistent with a developmental account describing a correlation between the integrity of frontostriatal white matter tracts and the ability to delay gratification (Achterberg et al., 2016), a finding with direct relevance for delay discounting behavior and the proposal that treatment-mediated increases in such control can improve clinical outcomes.

This study does come with limitations. Regression to the mean is a potential concern for studies that use crossover designs, and we have taken a number of steps to minimize that possibility. To start, the change in the impulsive choice ratio (ICR) was compared not to the baseline ICR, but to an independent measure, the non-planning subscale of the Barratt Impulsiveness Scale (BIS). Moreover, the fact that the significant correlation with the BIS non-planning subscale was specific to this measure – i.e. the same correlations with the BIS attention and BIS motor subscales were not significant – argues for a neurobiological basis rather than one determined by noise. In addition, we directly assessed the possibility of regression to the mean relative to the baseline ICR itself. As noted by Schmaal and colleagues in a similarly designed study (Schmaal et al., 2013), regression to the mean would be expected to result from session effects, rather than the effects of drug condition, as drug was counterbalanced across sessions. We therefore directly correlated the ICR in the first session with the change in ICR from session 1 to session 2. This correlation was not significant (r = − 0.26, p = 0.31). As Schmaal and colleagues also point out, if regression to the mean is present, the correlation between ICR in session 1 and ICR in session 2 should be minimal. In contrast, the correlation between sessions in our data was highly significant (r = 0.95, p ≪ 0.001). Thus, these findings do not support a substantial contribution from regression to the mean.

Other limitations were less easily addressed. The study size was itself relatively limited, due to a number of factors including the expenses related to the multiple MRI sessions and the study drug, and the need to ensure reliable subject attendance across three study visits separated by many days. Moreover, despite the fact that subjects underwent urine toxicology screening and breathalyzer testing prior to every visit, we cannot rule out the possibility that intervening use between our two study sessions may have confounded results. In particular, a positive screen for marijuana on urine toxicology screening can be seen for up to some weeks following last use, and our self-report measures about marijuana cessation at least 48 h prior to study visits could be unreliable if subjects were consistently untruthful during screening and subsequent visits. Similarly, while we relied upon previous work demonstrating that delay discounting behavior correlates with real-world addictive behaviors (Bickel et al., 2014), our study design did not permit us to determine whether tolcapone had effects on real-world gambling in these subjects. Longitudinal studies of tolcapone use would be much better positioned to address such questions.

A related concern has to do with the lack of a main effect of tolcapone on delay discounting behavior in this study cohort. Given the known heterogeneity in dopamine signaling across individuals (reviewed in (Cools and D'Esposito, 2011)), we did not expect to see a significant main effect of drug, as discussed above. However, we were also limited in our ability to identify those subjects a priori who might respond to tolcapone. In the future, better understanding the behavioral and neural factors that predict response to tolcapone and other dopaminergic drugs, potentially including the BIS and activity within the RIFC, would permit us to enrich our subject pool before the medication intervention for those felt to have the greatest likelihood of benefit.

Despite these limitations, these data argue that tolcapone may have a role in reducing impulsive behaviors in subjects with PPG, specifically via increases in top-down control in those individuals for whom baseline RIFC activity is greater. Future work in larger numbers of subjects to determine whether activity within the RIFC may be a potential biomarker for treatment response, and to link these laboratory assays to real-world clinical outcomes, would be critical next steps. From a broader perspective, such future work might help to fulfill the promise of precision medicine (Jameson and Longo, 2015) to develop new therapies for problem and pathological gambling.

## Funding and disclosure

This research was supported by funding from the National Center for Responsible Gaming (A.S.K.), the Institute for Molecular Neuroscience (grant W81XWH-11-2-0145 to A.S.K.), the Wheeler Center for the Neurobiology of Addiction (J.M.M.), and the Deutsche Forschungsgemeinschaft (grant PE1627/5-1 and TR CRC 134 project C05 to J.P.). The funders had no role in the conduct of the study or in preparation of the results for publication. The authors declare no conflicts of interest. This study is registered at http://clinicaltrials.gov under NCT #02772978.

## Figures and Tables

**Table 1:** Demographic and gambling-related data for study participants. Note that study subjects were not limited to identifying a single gambling activity. Abbreviations: AUDIT = Alcohol Use Disorders Identification Test. BIS = Barratt Impulsiveness Scale. LOC = Rotter's Locus of Control Scale. STPI – Future = Stanford Time Perspective Inventory, Future subscale. SOGS = South Oaks Gambling Scale. GRCS = Gambling-Related Cognitions Scale. GSAS = Gambling Symptom Assessment Scale (administered on both placebo and tolcapone study days). SCI-PG = Structured Clinical Interview for Pathological Gambling.

		Mean/Count	Range/Percentage
Age		31.5 ± 8.9	20–47
Gender	Male	11	64.7%
	Female	6	35.3%
Ethnicity	Caucasian	10	58.8%
	Asian	6	35.3%
	Mixed	1	5.9%
AUDIT		14.9 ± 6.0	5–24
BIS		70.5 ± 8.9	50–88
LOC		12.4 ± 4.8	3–22
STPI – Future		29.3 ± 6.9	17–42
SOGS		10.5 ± 3.4	6–18
GRCS		94.5 ± 16.5	58–116
GSAS – Placebo		24.3 ± 7.2	14–40
GSAS – Tolcapone		23.9 ± 6.9	13–39
SCI-PG (pathological gambling)	Meets criteria	9	52.9%
	Does not meet criteria	8	47.1%
Gambling activities	Card games	8	
	Slot machines	7	
	Sports betting	4	
	Bingo/mah jongg	3	
	Online (not specified)	3	
	Lottery	2	
	Roulette	2	
	Dice games	1	

**Table 2:** Significant regions identified by fMRI in the analyses of Fig. 4, Fig. 5 (all p < 0.05, corrected for multiple comparisons). MNI coordinates indicate the center of mass for each cluster; F statistics and associated probability values are displayed for the peak voxel within each cluster.

Area–Neg.	MNI–X	MNI–Y	MNI–Z	# Voxels	F value	p Value
*Correlation, Want (Placebo) with ICR (Tolcapone – Placebo),*Fig. 4*A*						
R premotor cortex	33	6	48	37	28.39	0.000084
R inferior frontal cortex	43	− 8	28	31	16.89	0.00093

*Correlation, Want (Tolcapone) with ICR (Tolcapone – Placebo),*Fig. 4*B*						
R inferior frontal cortex	44	− 6	27	76	45.24	0.0000068
R premotor cortex	32	10	47	47	23.81	0.0002

*Correlation, Connectivity (Tolcapone – Placebo) with ICR (Tolcapone – Placebo),*Fig. 5						
R putamen	31	2	4	10	17.84	0.00074

## Appendix A Supplementary data

Supplementary material demonstrating the main effect of task on tolcapone versus placebo. Image 1
