# Supplementary material for: Right inferior frontal cortex activity correlates with tolcapone responsivity in problem and pathological gamblers
Source: Neuroimage Clin. 2016 Dec 20;13:339–48. doi: 10.1016/j.nicl.2016.12.022 (PMC5200917; doi:10.1016/j.nicl.2016.12.022)
Supplement: Supplementary file 1 — Supplementary material demonstrating the main effect of task on tolcapone versus placebo. [file mmc1.docx]

Supplementary Figure 1:


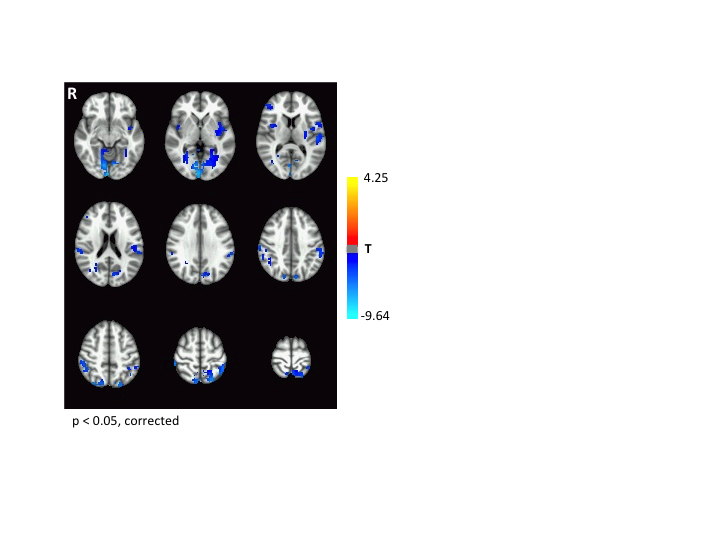


Main Effect of Task. Shown is a montage demonstrating the main effect of task on tolcapone versus placebo. The significant effects of tolcapone are found in cortical regions (see also Supplementary Table 1), most notably in parietal and visual cortices; and they indicate that BOLD activity declines on tolcapone. The relative absence of effects in prefrontal cortex is consistent with the absence of an overall group difference in delay discounting behavior (ICR) on tolcapone versus placebo.

Supplementary Table 1:

| Area–Neg. | MNI–X | MNI–Y | MNI–Z | # Voxels | T value | P value |
| --- | --- | --- | --- | --- | --- | --- |
| R lingual gyrus | 13 | 81 | -11 | 480 | -7.46 | 0.0000014 |
| L postcentral gyrus | -49 | 18 | 15 | 376 | -7.04 | 0.0000028 |
| L precuneus | -15 | 69 | 49 | 321 | -7.13 | 0.0000024 |
| L fusiform gyrus | -20 | 71 | -12 | 288 | -6.97 | 0.0000032 |
| R intraparietal lobule | 51 | 41 | 49 | 150 | -7.07 | 0.0000027 |
| R middle temporal gyrus | 30 | 62 | 9 | 95 | -9.64 | 4.6 x 10^-8^ |
| R precuneus | 16 | 75 | 51 | 94 | -6.96 | 0.0000032 |
| L intraparietal lobule | -42 | 48 | 59 | 81 | -6.45 | 0.000008 |
| R inferior frontal gyrus | 45 | -41 | 12 | 47 | -6.07 | 0.000016 |
| R postcentral gyrus | -64 | 23 | 38 | 44 | -5.40 | 0.000059 |
| R intraparietal lobule | 59 | 29 | 23 | 40 | -6.15 | 0.000014 |
| L precentral gyrus | -55 | -7 | 9 | 37 | -7.52 | 0.0000012 |
| R insula | 40 | -2 | 7 | 34 | -6.35 | 0.0000096 |

MNI coordinates for the contrast of the main effect of task on tolcapone versus placebo, as shown in Supplementary Figure 1 (p < 0.05, corrected for multiple comparisons). MNI coordinates indicate the center of mass for each cluster; T-values and p-values reflect results for the peak voxel in each cluster.
